# Small-sample antimicrobial resistance surveillance using lot quality assurance sampling in Uganda, Malawi, and Zambia

**DOI:** 10.1016/j.ijregi.2026.100988

**Published:** 2026-08-28

**Authors:** Robert Onzima Anguyo, Kennedy Uadiale, Phillip Chilembo, Joyce Chinyama Chilekwa, Elizabeth Ekirapa-Kiracho, Nicholas Feasey, Jerry Ictho, Angelo Lamadrid, Freddy Eric Kitutu, Henry Kajumbula, Katayi Kazimbaya, Peter Lochoro, Chrispus Mayora, Pius Mukisa, Charles Lubanga Nkolo, Thomasena O’Byrne, Marcello Pagano, Kapambwe Mwape, Michelo Simuyandi, Hannah Betts, Adam P. Roberts, Anjali Sharma, Joseph J. Valadez

**Affiliations:** 1 Liverpool School of Tropical Medicine, Pembroke Place, Liverpool, UK; 2 Doctors with Africa CUAMM, Kampala, Uganda; 3 Malawi Liverpool Wellcome Research Programme (MLW), Kamuzu University of Health Sciences, Blantyre, Malawi; 4 Centre for Infectious Disease Research in Zambia, Ibex Hill, Lusaka, Zambia; 5 Department of Community Health and Behavioural Sciences, Makerere University School of Public Health, Kampala, Uganda; 6 School of Medicine, University of St Andrews, St Andrews, UK; 7 Department of Health Care Management,Technische Universität Berlin, Berlin, Germany; 8 Department of Pharmacy, Makerere University School of Health Sciences, Kampala, Uganda; 9 Department of Women’s and Children’s Health, International Child Health and Migration, Uppsala University, Uppsala, Sweden; 10 Department of Microbiology, Makerere University School of Biomedical Sciences, Kampala, Uganda; 11 Biostatistics Department, Harvard T.H. Chan School of Public Health, Boston, MA

**Keywords:** AMR prevalence, ESBL-producing *Escherichia coli*, ESBL-producing *Klebsiella pneumoniae*, Methicillin-resistant *Staphylococcus aureus*, LQAS

## Abstract

**Objectives:**

We evaluated lot quality assurance sampling (LQAS) adapted for antimicrobial resistance (AMR) surveillance (AMR-LQAS), a small-sample approach designed to classify district AMR prevalence against a clinically meaningful resistance threshold in Uganda, Malawi, and Zambia.

**Methods:**

The study was conducted across nine districts/locations, with each country including one urban, one peri-urban, and one rural district/location. Upper and lower resistance thresholds were 20% and 5%, respectively, with predefined misclassification probabilities (α = 0.05, β = 0.10), yielding a minimum sample size of 44 per district. In Uganda, classifications were compared with larger probability samples (n ≥ 240 in each of three districts). Organisms assessed included extended-spectrum beta-lactamase (ESBL)-producing *Escherichia coli* and *Klebsiella pneumoniae*, and methicillin-resistant *Staphylococcus aureus*. Antibiotics assessed were those routinely used in the study countries.

**Results:**

In Uganda, concordance between AMR-LQAS and probability-sample classifications was nine of 12 organism-district comparisons. Across antibiotic-district comparisons, concordance was 46/48, resulting in overall reliability of 92% (55/60 comparisons). Resistance to ciprofloxacin, gentamicin, and trimethoprim-sulfamethoxazole in ESBL-producing *E. coli* exceeded the 20% threshold in all districts, whereas meropenem resistance exceeded the threshold in only one district. Zambia had fewer antibiotics below resistance thresholds than Uganda and Malawi.

**Conclusion:**

AMR-LQAS reliably classified district-level resistance using substantially smaller sample sizes than conventional prevalence surveys, generating actionable subnational information to support antibiotic stewardship and surveillance in low-resource settings.

## Highlights

• Established lot quality assurance sampling (LQAS) reliability for rapid antimicrobial resistance (AMR) surveillance in resource-limited settings.
• Demonstrated the granularity of AMR-LQAS classifications differentiating AMR in urban, peri-urban, and rural settings.
• Results showed high reliability with misclassification within expected limits.
• AMR-LQAS integration could strengthen AMR surveillance and guide targeted stewardship.

## Introduction

In 2019, an estimated 1.27 million deaths were attributable to antimicrobial resistance (AMR), with approximately 20% occurring in sub-Saharan Africa [1,2]. While many bacteria are developing resistance to antibiotics, at least half of *Escherichia coli (E. coli)* and *Klebsiella pneumoniae (K. pneumoniae)* invasive isolates are estimated to become resistant to third-generation cephalosporins by 2030 [3]. The World Health Organization (WHO) classifies extended-spectrum beta-lactamase (ESBL)-producing Enterobacterales, particularly *E. coli* and *K. pneumoniae*, as critical pathogens presenting major public health threats, driven by severely limited treatment options and a sparse antibiotic development pipeline. Another priority pathogen, methicillin-resistant *Staphylococcus aureus* (MRSA), alone was responsible for over 100,000 AMR-attributable deaths globally in 2019 [4]. In addition to this mortality burden, bacterial AMR substantially strains already fragile health systems by prolonging hospitalization, increasing the risk of treatment failure, and escalating healthcare costs. In this context, strengthened surveillance, enshrined in the WHO's Global Action Plan on AMR [5] as a central pillar of the global response [6,7], remains critical for mitigating resistance by generating timely, reliable data to enable effective action, protect health systems, and contribute to achieving multiple sustainable development goals [6,7].

The WHO Global Antimicrobial Resistance Surveillance System (GLASS) provides standardized guidance for laboratory-based AMR surveillance using routine clinical microbiology data from sentinel sites, improving international comparability of national-level AMR trends and prevalence. However, its reliance on clinically indicated specimens may have limited feasibility in settings with resource-limited diagnostic capacity and low healthcare utilization [8,9]. In such settings, GLASS diagnostic sample-based AMR surveillance may both over-represent AMR prevalence where cultures are only performed after multiple rounds of antimicrobial therapy and underestimate it where diagnostic capacity is limited [8,9]. Additionally, the GLASS guidelines do not provide structured district-level program decision rules and, along with incomplete subnational disaggregation found in many African settings, leave district-level health managers without actionable AMR information [10,11].

Furthermore, the COVID-19 pandemic disrupted AMR surveillance and stewardship activities in low- and middle-income countries (LMICs) due to decreased funding, highlighting the fragility of these surveillance systems to external shocks [12]. These limitations underscore the need for complementary surveillance approaches as recommended by the WHO [12]. Such approaches should generate reliable, decision-oriented information at district level using feasible sample sizes, particularly given the significant subnational heterogeneity in resistance patterns that directly guides empirical treatment and procurement decisions.

In this study, we evaluated a small-sample, decision-focused surveillance approach, AMR-LQAS (lot quality assurance sampling adapted for antimicrobial resistance), to classify districts against a clinically meaningful AMR prevalence threshold, with particular emphasis on resistance patterns involving ESBL-producing Enterobacterales and MRSA. Developed in the 1920s for industrial quality control and adapted for public health monitoring in the 1980s [13,14], LQAS uses small samples to classify geographic areas against predefined performance thresholds with explicit misclassification probabilities rather than measuring prevalence; for example, in a child health program with a district coverage target for on-time Diphtheria, Pertussis, Tetanus third dose (DPT3) vaccination of 85%, LQAS would classify the district’s probability of having achieved this target, requiring a smaller sample size than a prevalence survey and thereby improving operational efficiency, feasibility, and cost-benefit [15].

While LQAS has been applied to classify healthcare facilities according to resistance thresholds using routine diagnostic results or in simulation using routine AMR surveys [16,17], those applications relied on opportunistic diagnostic results from facility-based samples. Also, while comparative studies of health services coverage using LQAS and probability-based surveys such as Demographic and Health Surveys have demonstrated acceptable agreement [18], they too relied either on opportunistic laboratory specimens or simulation results. This adaptation of LQAS for district-level AMR classification contributes to LQAS extensions by using random population-based sampling with laboratory-confirmed resistance outcomes. It was then used for microbiological AMR surveillance across multiple countries.

We therefore adapted LQAS for district-level AMR surveillance (AMR-LQAS) among febrile children and evaluated its reliability against healthcare facility catchment-population-weighted prevalence estimates derived from larger probability samples per district in Uganda, followed by implementation in Malawi and Zambia to assess its feasibility and suitability for recurrent use across diverse urban, peri-urban, and rural districts.

## Methods

### Study design

This was a descriptive cross-sectional survey. We adapted LQAS, a decision-oriented sampling approach widely used in public health [19,20], for district-level AMR surveillance among febrile children aged under 5 years. LQAS prioritizes classification against predefined thresholds rather than precise prevalence estimation and is designed to support rapid program decision-making [15]. Classic LQAS classification uses a fixed sample size and a corresponding decision rule, which is the minimum number of cases showing resistance to classify a district with an unacceptably high prevalence of resistance; otherwise, the district is classified in the low resistance category. Designs require three decisions: upper and lower resistance thresholds for classifying districts, error terms for the classifications, and a statistically determined decision rule.

We first implemented AMR-LQAS in three districts of the Acholi sub-region of northern Uganda (urban Gulu City, peri-urban Gulu District, and rural Lamwo District) and later in Malawi and Zambia. In Malawi, the study was conducted in rural Chikwawa District and in Blantyre urban and Blantyre peri-urban within Blantyre District. Similarly, in Zambia, the study was conducted in Lusaka District (urban), Chilanga District (peri-urban), and Chongwe District (rural). In each district, all functioning eligible lower-level public and not-for-profit health facilities were included. Within each facility, consecutive eligible febrile children aged under 5 years were enrolled, which approximates systematic sampling where arrival order is not expected to correlate with resistance risk. Characteristics of the sampled children can be found in Table 1.

**Table 1:** Demographics of participants across Uganda, Malawi, and Zambia.

Child’s sex	Uganda		Malawi		Zambia		Overall	
Male	393	54%	102	60%	79	51%	574	55%
Female	331	46%	67	40%	77	49%	475	45%
**Child’s age category**								
0-11 months	280	39%	54	32%	60	39%	394	38%
12-23 months	169	23%	50	30%	38	24%	257	24%
24-59 months	275	38%	65	38%	58	37%	398	38%

### Design parameters

Design parameters were specified a priori with an upper threshold of 20% AMR prevalence for classifying a district as having high AMR prevalence and a lower threshold of 5% AMR prevalence. The 20% resistance threshold was selected as a programmatically meaningful level commonly used in empirical treatment guidance to trigger review of first-line antibiotic regimens [21]. Maximum allowable misclassification errors were set at *α* = 0.05 (false-positive classification) and *β* = 0.10 (false-negative classification). Under these specifications, an LQAS design with a minimum sample size of *n* = 44 and decision rule *d* = 5 was selected. Districts with five or more children carrying resistant bacteria or antibiotic-specific resistance in the sample were classified as reaching or exceeding the 20% threshold based on binomial probabilities. The prevalence of carriage of resistant bacteria and antibiotic resistance was calculated using the total sample of children as the denominator, rather than only those carrying the bacteria under investigation. We assessed multidrug resistance (defined as non-susceptibility of a pathogen to at least one antibiotic agent in three or more classes of antibiotics) [22] among ESBL-producing bacteria across five antibiotic classes.

Decision accuracy at selected prevalence levels is shown in Table S1 in the appendix as part of a sensitivity analysis. Full operating characteristics (Figure S1) are provided in the appendix and in a previous publication [14].

A further parameter was that the same number of children should be selected in each participating health facility to reduce possible non-sampling error resulting from data collectors sampling incorrect numbers of children in a health facility, with a minimum of six febrile children sampled in any health facility, the standard sample for an accompanying health facility assessment [23]. Because sample size, the decision rule, and α and β errors are interrelated [14], this additional parameter resulted in increased sample sizes for each research district (Table S2). The decision rule in Uganda for the sample sizes of *n* = 66, 72, and 66, respectively, for urban, peri-urban, and rural sites was *d* = 7 with corresponding error terms (*n* = 66, *α* = 0.014, *β* = 0.046; *n* = 72, *α* = 0.006, *β* = 0.068).

### Reliability assessment

Concordance was defined as agreement between the AMR-LQAS and probability-sample classifications for an individual comparison; overall reliability was defined as the proportion of all comparisons that were concordant.

To assess reliability, AMR-LQAS classifications in Uganda were compared with weighted district-level prevalence estimates derived from larger probability samples (*n* ≥ 240 per district) across four organism categories and three districts. These probability samples were designed to estimate prevalence with 95% confidence intervals. Comparisons within 5 percentage points around a 20% prevalence estimate were considered concordant (Tables 2, 3, S3, and S4). The rule used for this reliability study was that, for districts classified in the high category (Above), the probability-sample point estimate needed to be in the range 15-20% or higher than the 20% threshold to be concordant with the LQAS classification. For districts in the low category (Below), concordance was judged when the reliability sample point estimate was less than 15% (Tables 2, 3, S3, and S4).

### Target population and specimen collection

The principal target population comprised febrile children aged under 5 years attending lower-level health facilities. From each enrolled child, one nasal swab and one rectal swab were collected for culture of MRSA and ESBL-producing organisms (*E. coli* and *K. pneumoniae*) respectively, using chromogenic agar.

Nasal cultures from which MRSA strains were isolated were further assessed for resistance to erythromycin, gentamicin, tetracycline, and trimethoprim-sulfamethoxazole (SXT). Similarly, ESBL-producing *E. coli* and *K. pneumoniae* isolated from rectal swabs were further assessed for resistance to chloramphenicol, ciprofloxacin, gentamicin, meropenem, and SXT.

### Quality assurance and data management

Field teams underwent standardized training in informed consent, sampling, specimen collection, handling, and electronic data recording prior to data collection. Specimens were transported daily to designated national laboratories for culture and susceptibility testing according to standardized protocols with disk diffusion. Data were anonymized and analyzed using Stata version 19.

## Results

### Participant demographics

A total of 1,049 children were enrolled across Uganda (*n* = 724), Malawi (*n* = 169), and Zambia (*n* = 156); 574 (55%) were male and 475 (45%) were female; 394 (38%) of the children were 0-11 months old, 257 (24%) were 12-23 months old, and 398 (38%) were 24-59 months old (Table 1).

### Uganda and reliability assessment

Concordance of ESBL *E. coli*, ESBL *K. pneumoniae,* and MRSA carriage AMR-LQAS classifications using a 20% AMR threshold and prevalence estimates was observed in 9/12 comparisons (Table 2). Across antibiotic-specific classifications by organism in three districts (*n* = 48 district-antibiotic comparisons), concordance with the probability-sample estimates was 46/48 (Tables 3, S3, and S4). Overall reliability of AMR-LQAS classifications was 92% (55/60 comparisons with the probability sample).

**Table 2:** Agreement between AMR-LQAS district classification and weighted carriage prevalence estimates in Uganda.

Organism	District	LQAS sample (*n*)	Bacterial isolate present (*n*)	LQAS classification^a^	Probability sample	Weighted prevalence % (95% CI)	Agreement^b^
ESBL *Escherichia coli*	Gulu City	66	53	Above	242	76% (68-83)	Concordant
	Gulu District	72	57	Above	240	74% (67-80)	Concordant
	Lamwo	66	51	Above	242	78% (72-83)	Concordant
ESBL *Klebsiella pneumoniae*	Gulu City	66	7	Above	242	16% (10-23)	Concordant
	Gulu District	72	6	Below	240	15% (10-21)	Discordant
	Lamwo	66	5	Below	242	10% (7-15)	Concordant
MRSA	Gulu City	66	5	Below	242	6% (3-10)	Concordant
	Gulu District	72	9	Above	240	9% (5-14)	Discordant
	Lamwo	66	7	Above	242	6% (4-10)	Discordant
Any ESBL or MRSA organism	Gulu City	66	58	Above	242	82% (74-88)	Concordant
	Gulu District	72	57	Above	240	80% (73-85)	Concordant
	Lamwo	66	54	Above	242	81% (75-86)	Concordant
Overall agreement						9/12	

a Above threshold defined as ≥20% AMR prevalence using district-specific decision rules (see Methods).

b Concordance occurs when the Above classification and the prevalence estimate are in the range 15-20% or higher than the 20% threshold, or for the Below classification, when the prevalence estimate is <15%. AMR, antimicrobial resistance; CI, confidence interval; ESBL, extended-spectrum beta-lactamase; LQAS, lot quality assurance sampling; MRSA, methicillin-resistant *Staphylococcus aureus*.

**Table 3:** Agreement between AMR-LQAS district classification for ESBL *Escherichia coli* and weighted AMR antibiotic prevalence estimates in Uganda.

Antibiotic	District	LQAS sample (*n*)	Number resistant (*n*)	LQAS classification^a^	Probability sample (*n*)	Weighted prevalence % (95% CI)	Agreement^b^
Chloramphenicol	Gulu City	66	5	Below	242	11% (7-28)	Concordant
	Gulu District	72	6	Below	240	10% (7-15)	Concordant
	Lamwo	66	8	Above	242	10% (7-15)	Discordant
Ciprofloxacin (Watch)	Gulu City	66	42	Above	242	54% (45-62)	Concordant
	Gulu District	72	37	Above	240	53% (45-60)	Concordant
	Lamwo	66	39	Above	242	57% (50-63)	Concordant
Gentamicin	Gulu City	66	15	Above	242	26% (19-34)	Concordant
	Gulu District	72	17	Above	240	20% (14-26)	Concordant
	Lamwo	66	13	Above	242	17% (12-22)	Concordant
Meropenem (Watch)	Gulu City	66	1	Below	242	0% (0-1)	Concordant
	Gulu District	72	0	Below	240	0% (0-1)	Concordant
	Lamwo	66	0	Below	242	2% (1-5)	Concordant
SXT	Gulu City	66	47	Above	242	72% (64-79)	Concordant
	Gulu District	72	43	Above	240	61% (54-68)	Concordant
	Lamwo	66	47	Above	242	63% (57-69)	Concordant
Multidrug resistant ESBL *E. coli*	Gulu City	66	51	Above	242	74% (66-88)	Concordant
	Gulu District	72	49	Above	240	71% (64-78)	Concordant
	Lamwo	66	49	Above	242	74% (68-79)	Concordant
Overall Agreement							17/18

a Above threshold defined as ≥20% AMR prevalence using district-specific decision rules (see Methods) or with an estimated prevalence within 5 percentage points below 20%.

b Concordance occurs when the Above classification and the prevalence estimate are in the range 15-20% or higher than the 20% threshold, or for the Below classification, when the prevalence estimate is <15%. AMR, antimicrobial resistance; CI, confidence interval; ESBL, extended-spectrum beta-lactamase; LQAS, lot quality assurance sampling; SXT, trimethoprim-sulfamethoxazole.

All three districts were classified as exceeding the 20% threshold for ESBL *E. coli* carriage, consistent with weighted prevalence estimates ranging from 74% to 78%. For ESBL *K. pneumoniae* carriage, only Gulu City was classified as above the threshold, while only Gulu District had a discordant classification of Below the threshold.

For MRSA carriage, Gulu District and Lamwo District were classified as above the threshold despite weighted prevalence estimates below 10%, resulting in two discordant classifications (Table 2).

At the antibiotic level, carriage of resistance to ciprofloxacin, gentamicin, and SXT for ESBL *E. coli* exceeded the 20% AMR carriage prevalence threshold in all study districts in Uganda, whereas chloramphenicol resistance carriage was above 20% threshold, but was discordant with the weighted prevalence in Lamwo District. Meropenem resistance carriage remained below the threshold in all three districts. All three districts exceeded the 20% threshold for ESBL-producing *E. coli* multidrug resistance for antibiotics tested for both the reliability survey and the AMR-LQAS classifications. In contrast, Gulu and Lamwo districts were below this threshold for ESBL *K. pneumoniae* multidrug resistance (Tables 3, S3, and S4).

### Classifications of antibiotics by level of resistance in three countries

Across the three countries, all districts were classified in the high category reaching the 20% resistance carriage prevalence threshold for ciprofloxacin, gentamicin, and SXT for ESBL *E. coli* infections (Tables 4 and 5). In contrast, meropenem resistance remained in the low category with only one of nine districts classified in the high AMR prevalence category. For ESBL *K. pneumoniae*, meropenem and chloramphenicol resistance prevalence did not reach the 20% threshold for any district, indicating preserved susceptibility across the districts. For MRSA, resistance to gentamicin and tetracycline remained below the 20% threshold in all districts, which may indicate low carriage of MRSA resistance to these antibiotics. Resistance to erythromycin and SXT reached the 20% threshold in one and two of nine districts, respectively (Tables 4 and 5). These classifications identify antibiotics for which treatment effectiveness is likely to be preserved or compromised across districts, providing information relevant to regional treatment guidance.

**Table 4:** Number and percentage of districts classified as having high prevalence of antibiotic resistance carriage in nine districts across three countries (Uganda, Malawi, Zambia).

Antibiotic	ESBL *Escherichia coli* (*n* = 9 districts)	ESBL *Klebsiella pneumoniae* (*n* = 9 districts)	MRSA (*n* = 9 districts)
Chloramphenicol	2/9 (22%)	0/9 (0%)	..
Ciprofloxacin	9/9 (100%)	3/9 (33%)	..
Gentamicin	9/9 (100%)	2/9 (22%)	0/9 (0%)
Meropenem	1/9 (11%)	0/9 (0%)	..
SXT	9/9 (100%)	4/9 (44%)	2/9 (22%)
Erythromycin	..	..	1/9 (11%)
Tetracycline	..	..	0/9 (0%)

“..” indicates the antibiotic not assessed for an organism. ESBL, extended-spectrum beta-lactamase; MRSA, methicillin-resistant *Staphylococcus aureus*; SXT, trimethoprim-sulfamethoxazole.

Country-level results (Table 5) highlight variation in AMR patterns that is masked in regional summaries and may inform national treatment policy and health system management. Uganda and Zambia each had four antibiotics for which MRSA resistance remained below the 20% threshold, whereas Malawi had two.

**Table 5:** Number of district classifications reaching the 20% AMR prevalence threshold.^a^

Antibiotic by organism	Uganda			Malawi			Zambia			#reaching 20% AMR thresholds by #tests (%)	
	ESBL *Escherichia coli*^a^	ESBL *Klebsiella pneumoniae*	MRSA	ESBL *E. coli*	ESBL *K. pneumoniae*	MRSA	ESBL *E. coli*	ESBL *K. pneumoniae*	MRSA		
Chloramphenicol	1	0	..	0	0	..	1	0	..	2/18	11%
Ciprofloxacin	3	1	..	3	0	..	3	2	..	12/18	67%
Gentamicin	3	0	0	3	0	0	3	2	0	11/27	41%
Meropenem	0	0	..	0	0	..	1	0	..	1/18	6%
SXT	3	1	0	3	1	2	3	2	0	15/27	56%
Erythromycin	..	..	0	..	..	1	..	..	0	1/9	11%
Tetracycline	..	..	0	..	..	0	..	..	0	0	0%

“..” indicates the antibiotic not assessed for an organism.

AMR, antimicrobial resistance; ESBL, extended-spectrum beta-lactamase; MRSA, methicillin-resistant *Staphylococcus aureus*; SXT, trimethoprim-sulfamethoxazole.

a Three district tests for every country for each organism and antibiotic.

For ESBL-producing *E. coli*, Malawi had two of five antibiotics (meropenem and chloramphenicol), with resistance below the threshold in three districts while Zambia had two districts that were below the 20% threshold for these two antibiotics. Uganda had two and three districts with chloramphenicol and meropenem respectively below the threshold.

For ESBL-producing *K. pneumoniae*, Uganda and Malawi had a broader range of antibiotics with resistance below the threshold, although district variation was observed. In contrast, Zambia had only chloramphenicol and meropenem remaining below the resistance threshold.

## Discussion

This three-country validation study demonstrates that a small-sample, decision-focused AMR surveillance approach can reliably classify districts across diverse national and subnational settings against a clinically meaningful antibacterial resistance threshold with an overall reliability of 92%. Three-country analyses demonstrate heterogeneity in antibiotic resistance prevalence across districts.

While this is a validation study, in some districts, we note the suggestion of a striking return to chloramphenicol susceptibility among ESBL isolates.

High concordance with larger probability samples and antibiotic-level classifications suggests that AMR-LQAS can provide robust, district-level signals using substantially smaller sample sizes. Discordance between AMR-LQAS and the larger probability samples occurred in three organism-level comparisons in Uganda (ESBL-producing *K. pneumoniae* in Gulu District, and MRSA carriage in Gulu and Lamwo districts). By prioritizing AMR classification relative to a predefined prevalence threshold rather than precise prevalence estimation, AMR-LQAS addresses a key programmatic question: whether resistance in a defined population exceeds a level that should trigger changes in empirical treatment or procurement. In contrast, population-based prevalence surveys, while precise, require substantial laboratory capacity, large sample sizes, and extended field periods. Emerging approaches such as wastewater surveillance to monitor community-level resistance gene circulation remain difficult to interpret for clinical decision-making [24].

The resistance patterns observed in this study illustrate the utility of AMR-LQAS. Resistance to ciprofloxacin, gentamicin, and SXT for ESBL *E. coli* consistently exceeded the threshold we used across countries, whereas carbapenem (meropenem) resistance remained uncommon even in third-generation cephalosporin-resistant isolates. These patterns highlight the value of threshold-based surveillance in identifying both widespread resistance and preserved treatment options. Importantly, substantial heterogeneity across districts demonstrates how pooled regional summaries may mask important district-level differences. Zambia, for example, had fewer antibiotics remaining below the resistance threshold for ESBL-producing organisms than Uganda and Malawi. Such heterogeneity underscores the importance of subnational surveillance to inform tailored procurement, stewardship, and empirical prescribing decisions. In this regard, AMR-LQAS complements, rather than replaces existing systems such as GLASS in settings with weak laboratory infrastructure or low healthcare utilization by generating district-level classifications using feasible sample sizes and predefined error parameters [5,8]. Similarly, AMR-LQAS does not replace laboratory and hospital-based surveillance systems that are essential for monitoring pathogen-specific resistance trends and detecting emerging threats, particularly for invasive infections. Rather, AMR-LQAS supplements these existing systems by providing local-level threshold signals in settings where routine data are lacking or incomplete.

A key strength of AMR-LQAS is that it explicitly specifies acceptable misclassification probabilities, providing transparent operating characteristics that can support decentralized decision-making and local stewardship without requiring advanced statistical expertise [25]. Reduced sample size increases the feasibility of district-level AMR surveillance by lowering laboratory workload and logistical demands, thereby enabling more frequent and geographically extensive surveillance aligned with antibiotic initiatives, antibiotic distribution cycles, or guideline and policy review. For example, in the Uganda reliability study, the surveillance sample (*n* = 66) was more than three times smaller than the prevalence survey (*n* = 240). Also, prespecified error tolerances and decision rules allow district managers to interpret results without extensive statistical expertise, enhancing their decision-making capacity. Such decentralized decision-making is a recognized component of effective stewardship programs [26] and can contribute to localized improvements in LMICs [27], while informing national and regional health system policies even in post-conflict and post-disaster settings [28].

This study also has some limitations. First, surveillance focused on bacterial carriage among febrile children attending health facilities rather than invasive infection isolates (for example, MRSA isolated from a swab from a clinically apparent wound infection), which may limit direct inference to clinical outcomes. Second, being facility-based surveillance, this AMR-LQAS reflects resistance patterns among health facility users rather than the population at large. Third, consecutive enrollment within facilities may introduce selection bias if health facility attendance is systematically related to resistance risk. Fourth, the study evaluated a limited set of organisms and antibiotics because of available resource constraints. Fifth, while the 20% threshold is widely used in empirical treatment guidance, resistance decision points may vary across syndromes and settings. Despite the limitations, AMR-LQAS offers a scalable approach for health facility-level and subnational AMR surveillance in LMICs, with the potential to support community or district-level antibiotic stewardship, target empirical treatment review, and complement existing national and global surveillance platforms. Small-sample surveillance approaches may expand AMR monitoring in districts currently excluded from national reporting systems, thereby contributing to AMR reduction strategies [29].

AMR-LQAS provides a reliable, rapid surveillance approach that can be implemented by district-level health workers to guide antimicrobial stewardship. By explicitly linking decision points, sample sizes, and classification errors, and incorporating prespecified maximum error tolerances, AMR-LQAS can be adjusted to accommodate other decision thresholds. This multi-country study demonstrates the applicability of this small-sample approach in diverse settings in eastern and southern Africa with advantages over population surveys in ease and rapidity of implementation, speed of obtaining results, and utility for health systems management and policy change. Although this study demonstrates reliable classification against a resistance threshold, the extent to which such classifications alter empirical treatment guidelines, prescribing practices, or antibiotic consumption remains to be assessed. Future research should evaluate how district-level AMR-LQAS classifications influence antibiotic prescribing behavior, health system procurement decisions, and district stewardship over time. Linking AMR-LQAS results with routine antibiotic use in communities, facility-level prescribing audits, and pharmaceutical supply policies would enable evaluation of whether threshold-based signals translate into measurable AMR stewardship improvements. Cost studies of AMR-LQAS relative to other sampling approaches should also be planned to inform policy discussions, as has been done for the classic LQAS approach [15,18].

## CRediT authorship contribution statement

**Robert Onzima Anguyo:** Writing – review & editing, Writing – original draft, Validation, Supervision, Project administration, Methodology, Investigation, Formal analysis, Data curation, Conceptualization. **Kennedy Uadiale:** Writing – review & editing, Writing – original draft, Validation, Supervision, Project administration, Methodology, Investigation, Formal analysis, Data curation, Conceptualization. **Phillip Chilembo:** Writing – review & editing, Supervision, Investigation. **Joyce Chinyama Chilekwa:** Writing – review & editing, Writing – original draft, Validation, Supervision, Methodology, Investigation. **Elizabeth Ekirapa-Kiracho:** Writing – review & editing, Writing – original draft, Validation, Methodology, Conceptualization. **Nicholas Feasey:** Writing – review & editing, Writing – original draft, Validation, Methodology, Funding acquisition, Conceptualization. **Jerry Ictho:** Writing – review & editing, Methodology. **Angelo Lamadrid:** Writing – review & editing, Writing – original draft, Validation, Formal analysis. **Freddy Eric Kitutu:** Writing – review & editing, Formal analysis. **Henry Kajumbula:** Writing – review & editing, Methodology, Formal analysis, Conceptualization. **Katayi Kazimbaya:** Writing – review & editing, Investigation, Formal analysis. **Peter Lochoro:** Writing – review & editing, Supervision. **Chrispus Mayora:** Writing – review & editing, Methodology. **Pius Mukisa:** Writing – review & editing, Investigation, Formal analysis. **Charles Lubanga Nkolo:** Writing – review & editing, Investigation. **Thomasena O’Byrne:** Writing – review & editing, Supervision, Investigation. **Marcello Pagano:** Writing – review & editing, Validation, Formal analysis. **Kapambwe Mwape:** Writing – review & editing, Investigation, Formal analysis. **Michelo Simuyandi:** Writing – review & editing, Supervision, Investigation. **Hannah Betts:** Writing – review & editing, Writing – original draft, Validation, Investigation, Formal analysis, Data curation. **Adam P. Roberts:** Writing – review & editing, Writing – original draft, Funding acquisition. **Anjali Sharma:** Writing – review & editing, Writing – original draft, Validation, Supervision, Methodology, Funding acquisition, Conceptualization. **Joseph J. Valadez:** Writing – review & editing, Writing – original draft, Validation, Supervision, Methodology, Investigation, Funding acquisition, Formal analysis, Data curation, Conceptualization.

## Declaration of competing interest

The authors have no competing interests to declare.

## Appendix Supplementary materials

Image, application 1
