# Supplementary material for: Small-sample antimicrobial resistance surveillance using lot quality assurance sampling in Uganda, Malawi, and Zambia
Source: IJID Reg. 2026 Aug 28;21:100988. doi: 10.1016/j.ijregi.2026.100988 (PMC13631612; doi:10.1016/j.ijregi.2026.100988)
Supplement: Supplementary file 1 [file mmc1.docx]

**Appendix**

Contents

[Statistical design and operating characteristics of AMR-LQAS	1](#_Toc240459413)

[Design parameters	1](#_Toc240459414)

[Sensitivity analysis	2](#_Toc240459415)

[Table S1. Decision accuracy at policy-relevant thresholds	2](#_Toc240459416)

[Sampling framework and district implementation	3](#_Toc240459417)

[Health facility sampling framework	3](#_Toc240459418)

[Table S2. Local health facilities in three research districts per country (Uganda, Malawi, Zambia) with total surveillance and reliability sample sizes of nasal and rectal swabs from febrile children <5 years*	4](#_Toc240459419)

[Reliability assessment methods	5](#_Toc240459420)

[Training and data collection	5](#_Toc240459421)

[Antibiotic concordance	7](#_Toc240459422)

[Table S3. Agreement between AMR–LQAS district classification for ESBL *K. pneumoniae* and weighted AMR antibiotic prevalence estimates in Uganda	7](#_Toc240459423)

[Table S4. Agreement between AMR–LQAS district classification for MRSA and weighted AMR antibiotic prevalence estimates in Uganda	9](#_Toc240459424)

# Statistical design and operating characteristics of AMR-LQAS

## Design parameters

The parameters used for the model AMR-LQAS were:

- An upper resistance threshold of 20% resistance (pU=0·20); this was selected because 20% is a programmatically meaningful resistance level commonly used in empirical treatment guidance to trigger review of first-line antibiotic regimens.
- A lower resistance threshold of 5% (pL=0·05); this was selected as a programmatically meaningful sensitivity level and provides a feasible lower threshold to detect with a small sample.
- A maximum α error of 0·05 was used to minimise incorrect classification of current low-prevalence antibiotics as high, while a maximum β error of 0·10 minimized the risk of classifying antibiotics with high resistance as acceptable for treatment

The 20% resistance threshold reflects commonly used clinical decision guidance in antimicrobial stewardship, where empirical treatment regimens are typically reconsidered when resistance prevalence exceeds approximately 20%.

Using standard LQAS statistical formula for public health applications and URL<https://s3.eu-west-2.amazonaws.com/lqas.spectraanalytics.com/index.html>, a sample size and decision rule resulted (n=44, d=5) with an Operating Characteristic (OC) Curve demonstrating low probabilities of misclassification at the predefined 20% threshold. At a true prevalence of 20%, the probability of classification as high AMR is 0·956, corresponding to α=0·044. At a true prevalence of 5%, the probability of incorrect high classification is 0·068, corresponding to β=0·068 (Figure S1).

The OC Curve displays the probability of being classified in the high category for every AMR prevalence from 0-0·25 which approaches 100% at a prevalence of 0·25. LQAS was not developed to estimate prevalence precisely but rather to classify areas relative to predefined thresholds with known error probabilities, allowing reliable programmatic decisions using substantially smaller samples. The design therefore satisfies the predefined error constraints (α ≤0·05, β ≤0·10) for programmatic decision making.

Perhaps a simple explanation best captures the utility of AMR-LQAS. It is most precise in identifying HCFs with AMR prevalence away from the indifference region of the OC curve. At the rejection threshold of 0·20, HCFs are correctly classified as having high AMR prevalence 95·6% of the time. At prevalence ≥0·25, the probability of failing to identify an HCF as having high AMR approaches zero. HCFs with prevalence close to the rejection threshold may also have a high probability of rejection; for example, at a prevalence of 0·15, the probability of rejection is 0·810. Such classification is considered tolerable because the true prevalence is close to the 0·20 decision threshold and lies within the predefined tolerance region. Nevertheless, because the true prevalence of 0·15 is below the predefined 0·20 threshold, this is formally a false-positive classification.

## Sensitivity analysis

Sensitivity analyses confirmed robustness of classification performance near the 20% decision threshold (Table S1). An alternative parameter specification as used in Uganda (n=66, d=7) and detailed operating characteristics are also included in the sensitivity analysis in Table S1. Other samples size used in this study and their decision rules (n=60, d=6; n=48, d=5; n=49, d=5) displayed similar sensitivity (α=0·012, β=0·079; α=0·021, β=0·097; α=0·025, β=0·091, respectively).

| Table S1. Decision accuracy at policy-relevant thresholds | | | |
| --- | --- | --- | --- |
| True AMR prevalence | Probability classified as high AMR  (n=44, d=5) | Interpretation |  |
| 5% (lower threshold) | 0·068 | High confidence of correct negative classification |  |
| 10% (below threshold) | 0·453 | Moderate risk of false-positive |  |
| 15% (below threshold) | 0.810 | High risk of false positive |  |
| 20% (decision threshold) | 0·956 | High confidence of correct positive classification |  |
| 25% (above threshold) | 0·993 | Near-certain detection |  |
|  | (n=66, d=7) |  |  |
| 5% (lower threshold) | 0·046 | High confidence of correct negative classification |  |
| 10% (below threshold) | 0·494 | Moderate risk of false-positive |  |
| 15% (below threshold) | 0.884 | High risk of false positive |  |
| 20% (decision threshold) | 0·986 | High confidence of correct positive classification |  |
| 25% (above threshold) | 0·999 | Near-certain detection |  |

# Sampling framework and district implementation

## Health facility sampling framework

Lower-level health care facilities (HCF) were sampled because they are the most commonly used by communities for treatment of childhood illnesses. They typically are outpatient facilities within walking distance of surrounding communities. All eligible public and not-for-profit health facilities within the three research districts in each participating country were sampled with exclusion criteria being:

- specialised HCFs such those used for treating people with HIV/AIDS or family planning,
- institutional HCFs such as those for the military, police and prisons
- rural or peri-urban HCFs in urban districts, urban or rural HCFs in peri-urban districts, and urban or peri-urban HCFs in the rural districts
- having a very low caseload of children so as to prevent sampling six children in two days,
- nonfunctional facilities.

In each facility, in addition to taking nasal and rectal swabs to test for resistant bacteria and antibiotic resistance, clinicians treating febrile children who are experienced staff members of the HCF attending sick children on the day of the assessment were observed for quality of clinical care (QoC). This is a separate companion study of drivers of AMR. We sample children on a first come first serve basis, assuming statistical independence since febrile children arrive independently of each other. We exclude children referred to or out of the facility, and those referred for in-patient care as we cannot assess their QoC as the treatment protocol will differ and take place elsewhere, and not to delay their care. Children with a current or history of recurrent nose bleeding, those who received per-rectum treatment on the data collection day, and visitors to the research area were also excluded.

WHO guidance for cluster surveys recommends maintaining equal sample sizes in each cluster to simplify field implementation and reduce non-sampling error.^21^ The same guidance applies to this study. Accordingly, to reach the minimum of n=44 in each district and maintain the same sample size in each HCF, the total sample sizes differed between research areas (Table S2). To adhere to this WHO guidance, we targeted a minimum of six children per HCF. Depending on the number of eligible HCFs in each district, the number of children sampled per HCF was increased by one until the district-level sample size reached ≥44, the minimum required sample size per district – this explains the variation in the sample size between districts.

| **Table S2. Local health facilities in three research districts per country (Uganda, Malawi, Zambia) with total surveillance and reliability sample sizes of nasal and rectal swabs from febrile children <5 years*** | | | | |
| --- | --- | --- | --- | --- |
| Country | Urban | Peri-Urban | Rural | Row Totals in 3 districts |
| Uganda | Gulu City | Gulu District | Lamwo District | Totals |
| Number of Health Facilities | 11 | 12 | 11 | 32 |
| Total District Surveillance sample | 66 | 72 | 66 | 192 |
| District total reliability sample | 242 | 240 | 242 | 724 |
| Malawi | Blantyre Urban | Blantyre Peri-urban | Chikwawa District | Totals |
| Number of Health Facilities | 10 | 7 | 10 | 27 |
| Total District Surveillance sample | 60 | 49 | 60 | 169 |
| Zambia | Lusaka | Chilanga | Chongwe | Totals |
| Number of Health Facilities | 10 | 6 | 8 | 24 |
| Total District Surveillance sample | 60 | 48 | 48 | 156 |
| *Decision rules for n=48, 60, 66, 72 are 5, 6, 7, 7, respectively. | | | | |

# Reliability assessment methods

AMR-LQAS classifications were assessed for reliability using a probability sample, the approximate size of which was calculated using the formula n=1.96²pq/d² where p=0·20, q=0·0.80, n=245.89, d (precision)=±0·05 with 95% confidence (1·96). Uganda was selected as the site as it had the largest number of HCF. The resulting sample size (n=246) was to be sampled as a stratified random sample with the HCF being strata. Following WHO guidance recommending equal cluster sizes (strata in this case) to reduce field implementation errors. This advice obviates the need for data collectors to track which HCF needs a specific sample size. In this study Gulu City, Gulu District, and Lamwo had sample sizes of (22 x 11=242, 20 x 12=240 and 22 x 11=242) (as in Table S2) with 22 children sampled in each HCF in Gulu District and Lamwo, and 20 sampled in each HCF in Gulu District which resulted in 95% CI=±0·050, ±0·052, ±0·050, respectively. Weighted AMR prevalences were calculated using populations sizes in each HCF provided by the District Health Management Teams.

# Training and data collection

In each country, data were collected by two-person teams assigned to each health-care facility (HCF), with at least one team member being a clinician responsible for clinical observation and training the attending clinician at the HCF to collect nasal and rectal swab samples. If the attending clinician was uncomfortable collecting samples, the data collector clinician assumed responsibility for sample collection.

Data collectors were recruited from local Ministry of Health staff through the District Health Teams (DHTs), primarily from junior- to mid-level district health office personnel. This approach was intended to strengthen local capacity and facilitate learning about the proposed surveillance system, building on previous research demonstrating that the use of local staff to collect LQAS data is feasible and does not introduce systematic bias. A total of 20 data collectors participated in Uganda and 10 each in Malawi and Zambia. Each district had one district supervisor, and district-level supervision was conducted by three individuals per country.

Supervisors were individuals with management responsibilities within district health offices or health facilities and prior experience supervising health-related surveys. In all countries, training and oversight of data collection were conducted by the study investigators.

All data collectors and supervisors participated in a centralised 3 to 4-day training workshop, including one day of field practice. Training covered sampling procedures, interviewing techniques, specimen collection, and electronic data capture using Open Data Kit (ODK) on Android devices. Field practice allowed piloting of tools and procedures to identify and correct errors before field deployment.

Training was delivered through interactive methods, including lectures, group work, role plays, and field exercises, by experienced trainers from partner institutions led by the Liverpool School of Tropical Medicine. A microbiologist provided specialised instruction on nasal and rectal specimen collection. Data were captured electronically using ODK, and rectal and nasal swabs were stored at room temperature and transported for laboratory processing within 48 hours of collection.

Training and data collection dates in each country are as follows:

| Country | Training date (dd/mm/yy) | Data collection date (dd/mm/yy) |
| --- | --- | --- |
| Uganda | 28/04/25 - 30/04/25 | 05/05/25 - 16/05/25 |
| Malawi | 30/06/25 - 3/07/25 | 09/07/25 - 18/07/25 |
| Zambia | 30/09/25 - 3/10/25 | 6/10/25 - 17/10/25 |

## Antibiotic concordance

Tables S3 andS4 present organism-specific antibiotic classifications comparing AMR-LQAS results with weighted prevalence estimates from the probability sample. Comparisons with probability sample results within 5 percentage points around a 20% prevalence estimate were considered concordant.

| Table S3. Agreement between AMR–LQAS district classification for ESBL *K. pneumoniae* and weighted AMR antibiotic prevalence estimates in Uganda | | | | | | | | |  |
| --- | --- | --- | --- | --- | --- | --- | --- | --- | --- |
| Antibiotic | District | LQAS sample  (n) | Number  Resistant  (n) | LQAS * classification | Probability Sample  (n) | | Weighted Prevalence  % (95% CI) | Agreement † | |
| Chloramphenicol | Gulu City | 66 | 2 | Below | 242 | | 7% (4-14) | Concordant | |
|  | Gulu District | 72 | 4 | Below | 240 | | 6% (3-12) | Concordant | |
|  | Lamwo District | 66 | 3 | Below | 242 | | 2% (1-6) | Concordant | |
| Ciprofloxacin (Watch) | Gulu City | 66 | 7 | Above | 242 | | 15% (10-22) | Concordant | |
|  | Gulu District | 72 | 5 | Below | 240 | | 13% (9-18) | Concordant | |
|  | Lamwo District | 66 | 5 | Below | 242 | | 8% (5-13) | Concordant | |
| Gentamicin | Gulu City | 66 | 4 | Below | 242 | | 6% (3-11) | Concordant | |
|  | Gulu District | 72 | 1 | Below | 240 | | 8% (5-13) | Concordant | |
|  | Lamwo District | 66 | 2 | Below | 242 | | 2% (1-5) | Concordant | |
| Meropenem (Watch) | Gulu City | 66 | 0 | Below | 242 | | 0% | Concordant | |
|  | Gulu District | 72 | 0 | Below | 240 | | 0% | Concordant | |
|  | Lamwo | 66 | 0 | Below | 242 | | 0% | Concordant | |
| SXT | Gulu City | 66 | 7 | Above | 242 | | 14% (9-21) | Discordant | |
|  | Gulu District | 72 | 5 | Below | 240 | | 13% (9-19) | Concordant | |
|  | Lamwo District | 66 | 3 | Below | 242 | | 6% (4-10) | Concordant | |
| Multi-drug resistant ESBL *K. pneumoniae* | Gulu City | 66 | 7 | Above | 242 | | 15% (10-22) | Concordant | |
|  | Gulu District | 72 | 5 | Below | 240 | | 14% (10-20) | Concordant | |
|  | Lamwo District | 66 | 4 | Below | 242 | | 6% (4-10) | Concordant | |
| Overall Agreement | | | | | | 17/18 | | | |
| *Above threshold defined as ≥20% AMR prevalence using district-specific decision rules (see Methods) or with an estimated prevalence within 5 percentage points below 20%.  † Concordance occurs when the Above classification and the prevalence estimate are in the range (15-20%) or higher than the 20% threshold, or for the Below classification when the prevalence estimate is <15%. | | | | | | | | | |

| Table S4. Agreement between AMR–LQAS district classification for MRSA and weighted AMR antibiotic prevalence estimates in Uganda | | | | | | | | | | | | |  |
| --- | --- | --- | --- | --- | --- | --- | --- | --- | --- | --- | --- | --- | --- |
| Antibiotic | District | | LQAS sample  (n) | | Number Resistant  (n) | | LQAS classification | | Probability Sample  (n) | | Weighted Prevalence  % (95% CI) | Agreement † | |
| Erythromycin (WATCH) | Gulu City | | 66 | | 3 | | Below | | 242 | | 5% (3-9) | Concordant | |
|  | Gulu District | | 72 | | 3 | | Below | | 240 | | 5% (2-9) | Concordant | |
|  | Lamwo District | | 66 | | 1 | | Below | | 242 | | 2% (1-5) | Concordant | |
| Gentamicin | Gulu City | | 66 | | 4 | | Below | | 242 | | 2% (1-5) | Concordant | |
|  | Gulu District | | 72 | | 0 | | Below | | 240 | | 0% (0-2) | Concordant | |
|  | Lamwo District | | 66 | | 1 | | Below | | 242 | | 1% (0-2) | Concordant | |
| Tetracycline | Gulu City | | 66 | | 2 | | Below | | 242 | | 2% (1-4) | Concordant | |
|  | Gulu District | | 72 | | 5 | | Below | | 240 | | 4% (2-9) | Concordant | |
|  | Lamwo District | | 66 | | 3 | | Below | | 242 | | 3% (1-6) | Concordant | |
| SXT | Gulu City | | 66 | | 2 | | Below | | 242 | | 3% (1-7) | Concordant | |
|  | Gulu District | | 72 | | 5 | | Below | | 240 | | 4% (2-9) | Concordant | |
|  | Lamwo District | | 66 | | 2 | | Below | | 242 | | 2% (1-5) | Concordant | |
| Overall Agreement  Grand Total Agreement | |  | |  | |  | |  | | 12/12  55/60 (92%) | | |  |
| *Above threshold defined as ≥20% AMR prevalence using district-specific decision rules (see Methods) or with an estimated prevalence within 5 percentage points below 20%.  † Concordance occurs when the Above classification and the prevalence estimate are in the range (15-20%) or higher than the 20% threshold, or for the Below classification when the prevalence estimate is <15%. | | | | | | | | | | | | |  |

These results demonstrate close agreement between decisions made with AMR-LQAS district classifications and prevalence estimates derived from larger probability samples.
